# Genome-Wide Association Mapping of Crown and Brown Rust Resistance in Perennial Ryegrass

**DOI:** 10.3390/genes13010020

**Published:** 2021-12-22

**Authors:** Mattia Fois, Andrea Bellucci, Marta Malinowska, Morten Greve, Anja Karine Ruud, Torben Asp

**Affiliations:** 1 Center for Quantitative Genetics and Genomics, Aarhus University, 4200 Slagelse, Denmark; foismatt@qgg.au.dk (M.F.); and.be00@gmail.com (A.B.); m.malinowska@qgg.au.dk (M.M.); anja.ruud@qgg.au.dk (A.K.R.); 2 DLF Seeds A/S, 4660 Store Heddinge, Denmark; mg@dlf.dk

**Keywords:** perennial ryegrass, crown rust, brown rust, genome-wide association study, single nucleotide polymorphism

## Abstract

A population of 239 perennial ryegrass (*Lolium perenne* L.) genotypes was analyzed to identify marker-trait associations for crown rust (*Puccinia coronata* f. sp. *lolii*) and brown rust (*Puccinia graminis* f. sp. *loliina*) resistance. Phenotypic data from field trials showed a low correlation (*r* = 0.17) between the two traits. Genotypes were resequenced, and a total of 14,538,978 SNPs were used to analyze population structure, linkage disequilibrium (LD), and for genome-wide association study. The SNP heritability (*h^2^*_SNP_) was 0.4 and 0.8 for crown and brown rust resistance, respectively. The high-density SNP dataset allowed us to estimate LD decay with the highest possible precision to date for perennial ryegrass. Results showed a low LD extension with a rapid decay of *r*^2^ value below 0.2 after 520 bp on average. Additionally, QTL regions for both traits were detected, as well as candidate genes by applying Genome Complex Trait Analysis and Multi-marker Analysis of GenoMic Annotation. Moreover, two significant genes, *LpPc6* and *LpPl6*, were identified for crown and brown rust resistance, respectively, when SNPs were aggregated to the gene level. The two candidate genes encode proteins with phosphatase activity, which putatively can be induced by the host to perceive, amplify and transfer signals to downstream components, thus activating a plant defense response.

## 1. Introduction

Perennial ryegrass (*Lolium perenne* L.) is predominantly used as a forage crop in Europe, New Zealand, and temperate regions of Japan, Australia, South Africa, and South America due to its high productivity and high nutritional value in terms of palatability and digestibility [1]. As a turf, perennial ryegrass is extensively used in amenity lawns and sports grounds due to its excellent wear tolerance [2]. Perennial ryegrass is an outbreeding, mainly self-incompatible species, which reproduce by seed or asexually with new tillers’ production. Its relatively large genome (1C = 2.6 Gb) is assembled in 7 chromosomes (2n = 2x = 14) [3].

Plant growth, development, as well as productivity are adversely affected by biotic and abiotic stresses. Among the biotic stresses, crown rust, caused by the Basidiomycota fungus *Puccinia coronata* f. sp. *lolii*, is the most common and damaging disease in perennial ryegrass. The infection, common from July to October, is followed by sporulation, which causes several breaks on the leaf surface leading to increased transpiration, reduced water-soluble carbohydrate (WSC), and plant vigor. Crown rust infection can lower yield by up to 56%, along with reduced thousand-seed weight [4,5], negatively affecting perennial ryegrass seed production. Moreover, since both dry matter digestibility and efficient rumen fermentation are influenced by WSC concentration [6,7], crown rust infection likely affects both factors, reducing palatability and favoring saprophytes [8], which might threaten animal health.

Another rust pathogen belonging to the genus *Puccinia* is *P. loliina*, which is the causative agent of brown rust in perennial ryegrass. Despite the first description of brown rust dating back to the 1970s, there is very little information on its effect on perennial ryegrass. The disease occurs in spring and early summer [9], and whereas crown rust produces elongated yellowish pustules, brown rust appears with oval-shaped orange spores. However, similarity in the appearance of the two pathogens’uredial pustules can make the diagnosis of the disease difficult.

Resistance to crown rust in perennial ryegrass involves different mechanisms. Major genes, called resistance (*R*) genes, confer complete protection to a specific rust isolate, efficiently reducing pathogen growth [10]. Such resistance is controlled by one or a few genes, hence the name: qualitative resistance. However, this resistance mechanism has its limitations, mainly because *R* genes recognize only a limited number of pathogen races. A second mechanism involves quantitative trait loci (QTLs), where more genes with minor effects confer partial resistance with a certain degree of additivity [11], hence the name quantitative resistance. Studies support the idea that some quantitative resistance genes share structural and functional similarities with *R* genes belonging to the nucleotide-binding site and leucine-rich repeat (*NBS-LRR*) class [12,13]. Mechanisms involving multiple major and minor genes result in more durable disease control than mechanisms involving a single major gene [14].

Genetic markers, such as single nucleotide polymorphism (SNP), have proven to be useful for identifying genes/QTLs associated with important agronomic traits. Genome-wide association studies (GWAS) are extensively used to identify marker-trait associations for a range of agronomic traits in many crops. Association analysis is based on linkage disequilibrium (LD), defined as the non-random association of alleles at two loci [15]. A low LD decay was detected in perennial ryegrass [16,17]; moreover, Xing et al. [18] found a substantial LD decay within a physical distance of 500 bp for most considered disease resistance genes. Thus, to detect rust resistance candidate genes, a very dense marker set would be required. Therefore, whole-genome resequencing was performed in this study on a perennial ryegrass population phenotyped for crown and brown rust resistance.

There are several methods to perform association studies, and some of them are more suited for the analysis of large datasets, such as Genome Complex Trait Analysis (GCTA) [19] and Multi-marker Analysis of GenoMic Annotation (MAGMA) [20]. The first method relies on Restricted Maximum Likelihood (REML) analysis to estimate the proportion of additive genetic variance captured by all the SNPs, and it is mostly used in complex human disease studies. The MAGMA approach aggregate multi-marker effects to the whole gene’s level and then test the gene’s association with the studied trait. 

The aims of this study were (i) to resequence a diploid perennial ryegrass population to generate a high-density SNP marker set, (ii) to identify markers associated with crown rust resistance (CRR), and brown rust resistance (BRR) using genome-wide association mapping, and (iii) to identify candidate genes that potentially play a role in the resistance to the two diseases.

## 2. Materials and Methods

### 2.1. Plant Material and Phenotyping

The plant material was developed from 350 diploid populations selected as part of the Public-Private Partnership for pre-breeding in perennial ryegrass project by combining accessions and cultivars [21] (Figure S1). Ten seeds from each population were sowed out in two locations to reduce the effect of selection at DLF Seeds A/S (Store Heddinge, Denmark) and Graminor (Ås, Norway). The 3500 plants obtained per location were open-pollinated in 2013, and one seed from each plant was collected. All 3500 single-descendant seeds were sown in 2014 at the same location and open-pollinated in 2015. This time, two seeds per plant were harvested, and the seeds from the two locations were pooled to establish a broad base breeding population. 

During summer 2018, a collection of 1000 seeds was randomly selected from the broad base breeding population to be tested for crown rust resistance. The seeds were germinated, and the genotypes were cloned in two. One clone was established in a nursery at DLF Seeds A/S (55°18′02″ N; 12°24′51″ W), while the second was established in Les Alleuds (47°13′21″ N; 0°24′30″ W), France, for the rust infection trial.

The genotypes were tested once during summer 2018 in two replicates placed side by side, giving one average observation. Rust infection was scored visually on a scale from 1 (heavy attack) to 9 (no rust infection). The genotypes were scored for brown rust (BR) due to an early attack by *Puccinia loliina*. Later in the season, all genotypes were scored for crown rust (CR).

### 2.2. Core Collection

Genotypes’ best linear unbiased predictors (BLUPs) for CR and BR were estimated using the R package lme4 [22], including the genotype effect as fixed. Residuals were set as a random effect.

BLUPs were used to select a representative subset using the R package Core Hunter 3 [23] with a core subset size set equal to 0.24 and the rest as default. Based on this analysis, a core collection of 240 out of 1000 genotypes were selected for genotyping. 

The Pearson’s product-moment correlations (*r*^2^) were estimated using BLUPs from BR and CR datasets to measure the strength of the linear relationship between the brown and crown rust measurements.

### 2.3. DNA Isolation and Sequencing

Genomic DNA was extracted from 240 clones grown in the nursery in Denmark using a modified version of the CTAB methods [24] and quantified by Quant-IT TM PicoGreen TM dsDNA assay kit (Invitrogen, Eugene, OR, USA). Library preparation and whole-genome resequencing were performed by Beijing Genomics Institute (Copenhagen, Denmark). The samples were sequenced to 15X genome coverage, assuming a genome size of 2.5 Gb as 150 bp paired-end sequencing on the BGISEQ-500 platform. One sample was discarded due to problems with poor DNA quality.

### 2.4. Genotyping, LD, and Population Structure

Quality control of the sequencing reads was performed using FastQC [25]. Adapter and low-quality bases were removed using Trim-galore [26], and BWA mem [27] was used to align the reads against the reference and generate an alignment file in SAM (Sequence Alignment/Map) format. Alignments were sorted by coordinate with Picard tools [28]. The Genome Analysis Tool Kit version 3.7 (GATK) [29] was used to generate a list of putative INDELs (RealignerTargetCreator), perform local realignment (IndelRealigner), and call SNPs (HaplotypeCaller and GenotypeGVCFs). The SNPs were filtered using GATK’s SelectVariant to obtain biallelic sites with minimum Mapping Quality of at least 30 and Minor Allele Frequency (MAF) of 0.03.

The function plink.prune [30] was used to generate a pruned subset of SNPs in approximate linkage equilibrium with each other, based on pairwise genotypic correlation setting the window size in SNPs as 50, the number of SNPs to shift the window at each step as 5, and the *r*^2^ threshold of 0.9.

Intra-chromosomal LD was investigated considering the whole, unpruned, genotypic dataset for each chromosome. All calculations were done using the PopLDdecay software [31]. First, pairwise LD between SNPs was calculated as *r*^2^ considering markers with a maximum distance of 50 Kbp, excluding SNPs with MAF below 0.03 and more than 20% of missing data points. Pairwise LD *r*^2^ values were assigned to bins depending on the distance between SNPs considered and the calculated mean *r*^2^. Finally, these values were used for plotting and evaluating LD decay as a function of genomic distance. 

Additionally, to analyze the population structure, principal component analysis (PCA) was performed on the pruned set of markers using the *-pca 20* flag from the GCTA package [19]. 

### 2.5. Genome-Wide Complex Trait Analysis (GCTA)

Genome-wide association mapping was conducted using the software package GCTA [19] based on REML statistics. A genetic relationships matrix (GRM) was calculated, adding the *-autosome* flag to the *-make-grm* flag, using the equation:

(1) $$A_{jk}=\frac{1}{N}\overset{N}{\underset{i=1}{\sum }}\frac{\left(x_{ij}-2p_{i}\right)\left(x_{ik}-2p_{i}\right)}{2p_{i}(1-p_{i})}$$

where A is the GRM between individuals j and k, over *N* SNPs, and i represents a specific SNP. $x_{ij}$ (coded as 0, 1, or 2) is the number of copies of the minor allele for the i SNP of the j individual. $p_{i}$ is the frequency of the minor allele for SNP i. 

Subsequently, estimations of the additive genetic variance and SNP heritability (*h*^2^_SNP_) were calculated using the GRM through the -*gcta64*-*reml* command with default settings. A log-likelihood ratio test was applied to test the significance of the estimated *h*^2^_SNP_.

The GRM was used to correct for population structure and relatedness among individuals during the GWAS analysis. Two different options were used to perform the association study, a mixed linear model association (MLMA) -GCTA, using the following model:

(2) y=a+bx+g+e

where y is the phenotype, a is the mean term, b is the additive effect (fixed effect) of the candidate SNP to be tested for association, x is the SNP genotype indicator variable coded as 0, 1, or 2, g is the GRM calculated using all the SNPs (random effect), and e is the residual. The second option, MLMA leaving-one chromosome-out (LOCO), implemented the MLMA with the chromosome on which the candidate SNP is located, excluded from calculating the GRM. Markers with a −log_10_ (*p*-value) > 5 were considered for further analysis. The results were visualized as Manhattan plots using the qqman R package [32].

### 2.6. Multi-Marker Analysis of Genomic Annotation (MAGMA)

A second approach to identify marker-trait associations for disease resistance was to use MAGMA v1.07b [20]. This analysis consists of two steps: annotation and gene analysis.

In the annotation step, SNP location (bp) and gene location (start and stop bp) were used to map SNPs onto gene regions by adding the nonhuman modifier to the annotation command.

The gene analysis step was performed on genotype data in PLINK format, to test the association between phenotype and SNP sets in the annotated genes. Gene *p*-values were computed under the *-snp-wise = mean* model, which uses the sum of −log_10_(*p*-value) of the SNPs as a test statistic to compute the gene *p*-value.

### 2.7. Annotation of QTL Regions

For GWAS results obtained by GCTA, genes located 5 Kbp before and after SNPs with −log_10_(*p*-value) > 5 were considered to identify functional genes associated with the two traits. For results from MAGMA, genes with −log_10_(*p*-value) > 4 were considered.

Gene Ontology (GO) and Interpro analysis were performed to identify their biological process, cellular component, and molecular function. Additionally, the selected candidate gene sequences were analyzed by BLAST-N against the NCBI database, and candidates with an identity (I) higher than 75% were considered.

## 3. Results

### 3.1. Phenotypic Analysis

**Figure 1:**
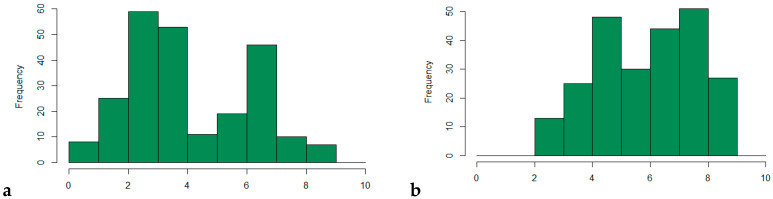
Distribution of the phenotypic scores among the 239 genotypes in the core collection for crown rust (**a**) and brown rust (**b**). The *x*-axis refers to the scale used for the disease scoring, where 1 refers to heavy attack (high susceptibility) and 9 corresponds to no rust infection (high tolerance).

Based on the phenotypic rust screening experiment, contrasting genotypes for rust response have been identified. The phenotypic data were distributed on the entire grading scale for the two traits (Figure 1). The core collection of 239 genotypes showed a bimodal distribution for both diseases with two modes at 3 and 7 for CR (Figure 1a) and 5 and 8 for BR (Figure 1b). The distribution of the initial 1000 genotypes showed a similar distribution (Figure S2).

To have a better understanding of the phenotypic variability of the selected genotypes, variance, standard deviation (SD), and coefficient of variation (CVar) were estimated (Table 1). The range of values presented a relatively higher dispersion around the mean, as described by the coefficient of variation in CR (48%) compared to BR (29%). Furthermore, trait repeatability was 0.70 and 0.78 for CR and BR, respectively. The correlation between BR and CR was low, with a value of *r* = 0.17 (*p*-value of 0.01), which means no significant correlation between the two traits.

### 3.2. Molecular Markers, LD and Population Structure

After filtering the SNPs, a total of 14,538,978 SNPs were used for subsequent analysis. Of these, 259,221 were scaffolds, while the rest were distributed on the seven chromosomes with an average of one SNP every 171 bp (Table 2). After pruning, the final marker set consisted of 9,045,818 SNPs and was used for the analysis. A high portion of rare alleles was identified from the Site Frequency Spectrum analysis when considering all the genotypes as a population (Figure S3). 

**Figure 2:**
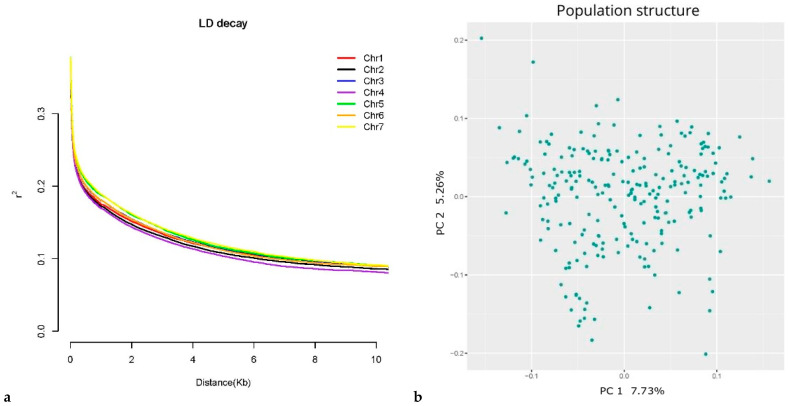
Genome-wide LD decay in the core collection population (**a**). LD decay is measured as a function of the distance between SNPs for each chromosome. The colour code is described in Figure 2a. Principal Component Analysis of population structure (**b**). The x- and *y*-axis refer to the first two principal components.

Intra-chromosomal LD, measured as *r*^2^, showed rapid decay below 0.2 after a few hundred base pairs, as shown in Figure 2a, with relatively high variation depending on the chromosome considered. Chromosome 4 showed the steepest LD decay with mean *r*^2^ > 0.2 after only 380 bp, followed by chromosome 2 (390 bp), chromosome 1 (430 bp), chromosomes 3 and 6 (520 bp), chromosome 5 (680 bp), and chromosome 7 (720 bp). When considering 0.1 as *r*^2^ value for LD decay, the trend was similar with distance values between 5.5 Kbp for chromosome 4 and 7.7 Kbp of chromosome 7 with an average LD decay below 0.1 at 6.8 Kbp.

The population structure analysis revealed a lack of clear stratification among the 239 genotypes (Figure 2b), with the first two principal components explaining a small portion of the variation in the data set, PC1 = 7.73% and PC2 = 5.26%. These results can be explained by the origin of the selected genotypes from two years of open pollination and selection aimed at developing a broad base breeding population.

### 3.3. Crown Rust Resistance Loci

There were no distinct differences between the results from the two GCTA options, MLMA and LOCO; for this reason, only results from MLMA are shown and through the text we refer to them as GCTA results. Additive genetic variance and *h^2^*_SNP_ were estimated, showing an *h^2^*_SNP_ of 0.4. The summary of the GCTA results is presented in Table 3. From the GWAS analysis performed with GCTA, none of the SNPs were statistically significantly associated with CRR after Bonferroni correction (−log_10_(*p*-value) > 7.72) (Figure S4a); thus, an arbitrary cutoff of −log_10_(*p*-value) > 5 was applied and a total of 60 SNPs above the chosen threshold were identified for CRR (Table S1). Chromosome 2 harboured the largest number of the SNPs above the threshold (20), whereas chromosome 5 had only one; the rest of the SNPs were equally distributed across the remaining chromosomes. 

Considering a region of 5 Kbp from each of the selected SNPs, 22 annotated genes were identified (Table S2). Integrating InterPro information with GO, relevant biological activities related to stress response were identified. 

**Figure 3:**
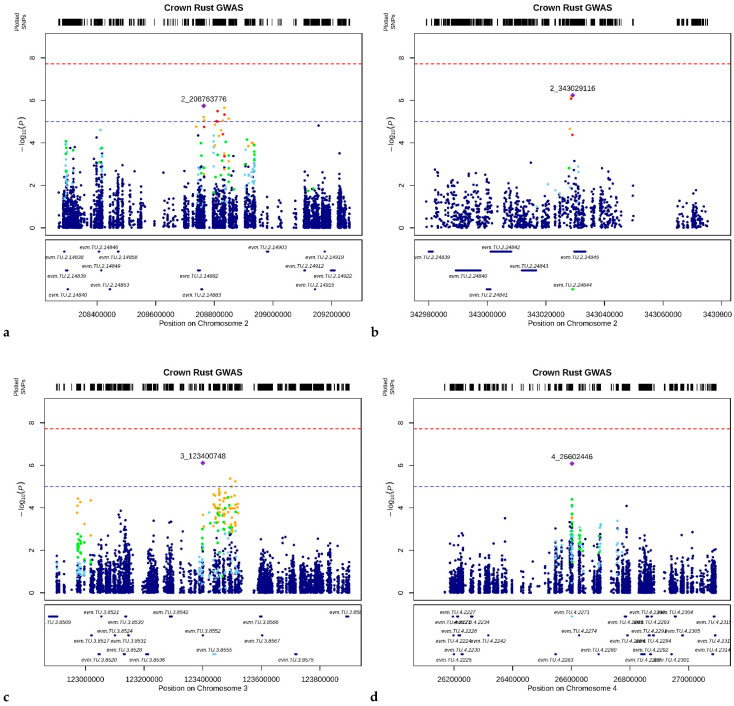

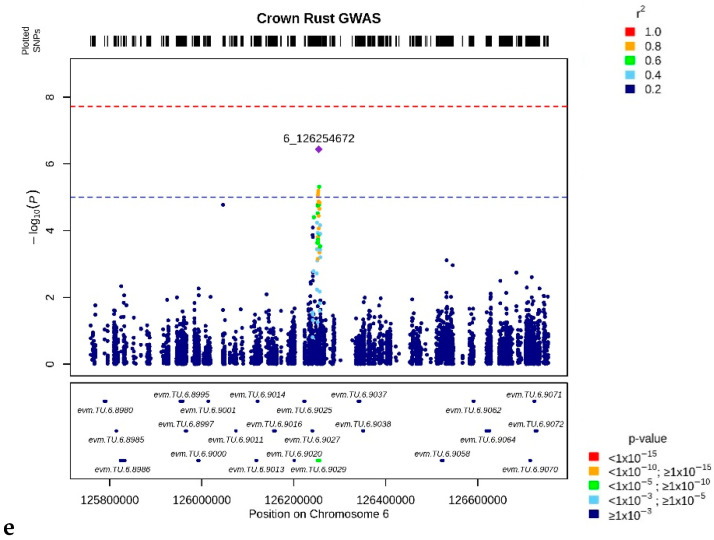
LocusZoom plots of the QTL associated with CRR from the GCTA analysis. Plots show the regions around the most significant SNPs. SNPs are plotted as the −log_10_ of the *p*-value (box on the top). The LD.Measure function from LDcorSV R-package [35] was used to estimate the linkage disequilibrium (*r*^2^) between the most significant marker in that region and all the markers within a 100 Mbp window; each SNP’s *r*^2^ is color-coded, as well as the *p*-value of the genes measured through MAGMA analysis (box on the bottom). The red dotted line refers to the Bonferroni threshold, while the blue horizontal line indicates the arbitrary cutoff of −log_10_(*p*-value) = 5. Each plot refers to the association of CRR to CR-QTL2.1 (**a**), CR-QTL2.2 (**b**), CR-QTL3 (**c**), CR-QTL4 (**d**), CR-QTL6 (**e**), respectively.

Five QTLs were discovered across the genome (Figure 3). On chromosome 2, a QTL, CR-QTL2.1, was detected at approx. 208 Mbp with 10 SNPs in close proximity over the arbitrary threshold (Figure 3a). Neither considering a region of 5 Kbp from each of the 10 SNPs nor through the MAGMA approach were detected any genes with a significant association with CRR. On the same chromosome, the QTL CR-QTL2.2, with three SNPs in high LD (*r*^2^ > 0.8), was identified at 343 Mb (Figure 3b). A gene, *evm.TU.2.2845*, was found in the 5 Kbp region. BLAST-N analysis revealed homology (I = 86.75%) with a transmembrane receptor protein kinase (TMK). Additionally, GO information revealed a biological process and a molecular function linked to the ubiquitin-dependent protein catabolic and protein binding processes. The MAGMA analysis *evm.TU.2.2845* showed a *p*-value > 1 × 10^−3^, whereas a second gene, *evm.TU.2.2844*, at only 300 bp distance from the previously mentioned gene, showed a stronger association with CRR (−log_10_(*p*-value) = 5.7). However, this gene had no GO and BLAST-N annotation.

On chromosome 3, the QTL CR-QTL3 was identified at 123 Mbp (Figure 3c), and a single gene, *evm.TU.3.8552*, was identified within the 5 Kbp region showing similarity (I = 91.08%) to a valine-glutamine (*VQ*) motif encoding gene in *Aegilops tauschii*, which previosly has been reported to be involved in disease response [33]. From the MAGMA analysis, the gene *evm.TU.3.8552* showed a *p*-value < 1 × 10^−3^, whereas the gene *evm.TU.3.855*, while being further than 5 Kbp from the three most significant SNPs of the QTL, showed a −log_10_(*p*-value) = 4.63. The *evm.TU.3.855* gene showed homology (I = 84.90%) with two genes involved in triterpene biosynthesis in *Avena strigosa* (*Sad1* and *Sad2*) through BLAST-N analysis, and a previous study showed their involvement in plant defense mechanisms [34]. 

On chromosome 4, at approx. 26 Mbp, CR-QTL4 was detected with only one SNP above the threshold (Figure 3d). A gene, *evm.TU.4.2271*, was identified using the MAGMA approach, within the 5 Kbp region, with a −log_10_(*p*-value) = 4.65. BLAST-N information showed homology (I = 87.01%) with a phytohormone transporter *NTR1/PTR* in *A. tauschii*, while GO annotation revealed a biological process linked to transmembrane transporter. 

**Figure 4:**
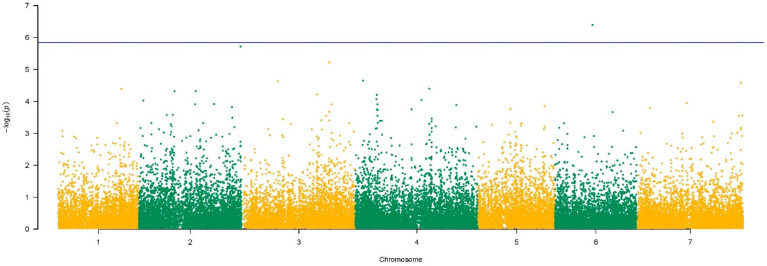
Manhattan plot of GWAS results for CRR using MAGMA. The blue line refers to the Bonferroni correction. On the *x*-axis, the 7 chromosomes are reported in alternating colours.

A QTL on chromosome 6, CR-QTL6, was identified at approx. 126 Mbp (Figure 3e). A single candidate gene was identified in the 5 Kbp region from the significant SNPs, *evm.TU.6.9020*, which was the only gene with a −log_10_(*p*-value) = 6.4 to pass the Bonferroni threshold in the MAGMA analysis (Figure 4). BLAST-N analysis showed an identity of 94% with a plastid casein kinase 2 gene (*CK2*) in *A*. *tauschii*. GO annotation revealed a molecular function related to protein kinases. 

The MAGMA analysis resulted in a higher number of candidate genes linked to biotic stress responses considering a threshold of −log_10_(*p*-value) > 4.0 (Table 4). BLAST-N information of the candidate genes identified by GCTA and MAGMA is listed in Table S3.

### 3.4. Brown Rust Resistance Loci

Results with GCTA analysis for BRR showed a relatively high *h^2^*_SNP_ (0.8) (Table 3). According to the Bonferroni threshold (−log_10_(*p*) = 7.72), none of the pruned SNPs was statistically significantly associated with BRR (Figure S4b); thus, an arbitrary cutoff of −log_10_(*p*-value) > 5 was applied. 

Results showed a total of 48 SNPs equally distributed on the seven chromosomes (Table S4), and 27 genes were detected in a region of 5 Kbp before and after each selected SNP (Table S5). Interpro and GO annotation reveal biological processes which might be related to response to biotic stimulus.

**Figure 5:**
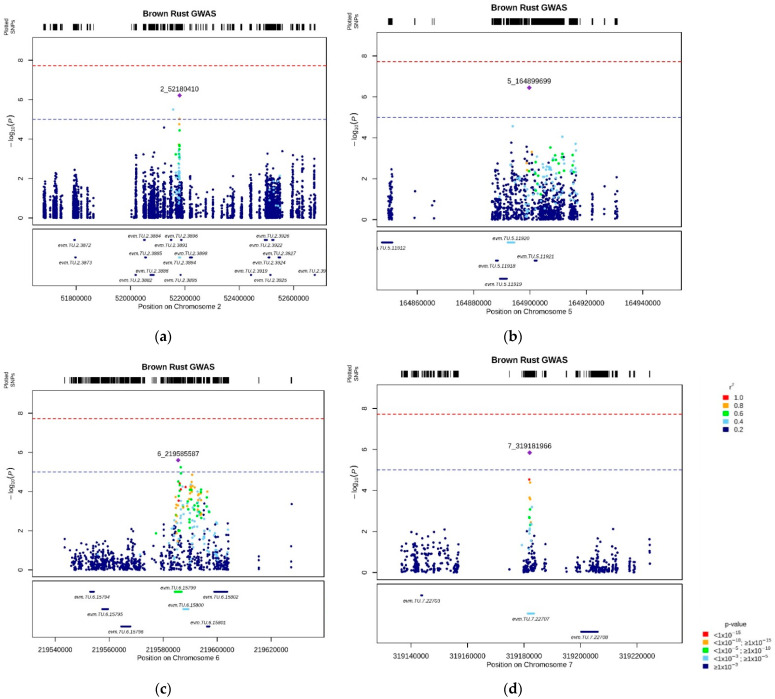
LocusZoom plots of the most highly associated QTL to BRR. Plots show the regions around the most significant SNPs. SNPs are plotted as the −log_10_ of the *p*-value (box on the top). The LD.Measure function from LDcorSV R-package [33] was used to estimate the linkage disequilibrium (*r*^2^) between the most significant marker in that region and all the markers within a 100 Mbp window; each SNP’s *r*^2^ is color-coded, as well as the *p*-value of the genes measured through MAGMA analysis (box on the bottom). The red dotted line refers to the Bonferroni threshold, while the blue horizontal line indicates the arbitrary cutoff of -log_10_(*p*-value) = 5. Each plot refers to the association of BRR to BR-QTL2 (**a**), BR-QTL5 (**b**), BR-QTL6 (**c**), BR-QTL7 (**d**), respectively.

Across the genome, four QTL regions were identified for BRR (Figure 5). On chromosome 2, the region BR-QTL2 was detected at 52 Mbp (Figure 5a). Two genes were identified in a region of 5 Kbp from the most significant SNP in the QTL region. The gene *evm.TU.2.3895* showed homology (I = 80%) to a gene encoding an S-narcoclaurine synthase (NCS) protein in *Brachypodium distachyon*, and its biological process is involved in response to biotic stimuli. However, the MAGMA analysis measured its *p*-value > 1 × 10^−3^, while the second gene, *evm.TU.2.3894*, showed a −log_10_(*p*-value) equal to 4.39. BLAST-N analysis of the second gene reported homology (I = 82%) with a gene encoding a Serine/arginine-rich protein from *B. distachyon*. 

A second QTL was identified on chromosome 5, BR-QTL5, at approx. 164 Mbp (Figure 5b). A gene in that region, *evm.TU.5.11920*, with a stronger association (−log_10_(*p*-value) = 4.22) to BRR, showed homology (I = 93%) with a gene from *A. tauschii* (I = 86.72) encoding a GTP-binding protein.

**Figure 6:**
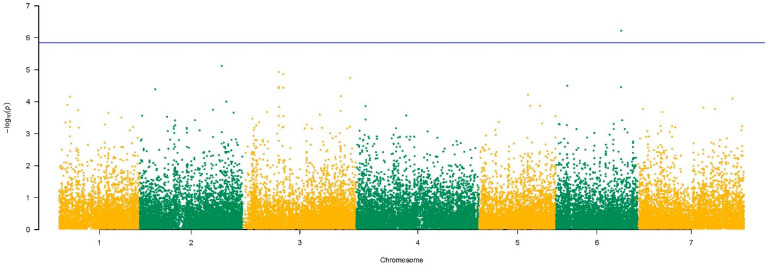
Manhattan plot of the GWAS results for BRR using MAGMA. The blue line refers to the Bonferroni threshold. On the *x*-axis, the 7 chromosomes are reported in alternating colours.

On chromosome 6, the QTL BR-QTL6 was detected at 219 Mbp (Figure 5c). Two genes were identified within a region of 5 Kbp from the QTL. The gene *evm.TU.6.15799*, with 56 SNPs within the gene sequence of 2507 bp, showed a −log_10_(*p*-value) equal to 6.22. According to the MAGMA results (Figure 6), this gene was the only one to show a significant association to BRR with a gene *p*-value above the Bonferroni threshold, −log_10_(*p*-value) = 5.8. BLAST-N analysis showed homology (I = 85%) to an inositol polyphosphate 5-phosphatase gene (*IP5PTase*) from *B. distachyon*, and GO information associated its biological function to phosphatidylinositol dephosphorylation. The second gene, *evm.TU.6.15800*, with a size of 1956 bp with 16 SNPs, showed a −log_10_(*p*-value) equal to 4.46. BLAST-N and GO information revealed its biological process linked to phosphatidylinositol transfer activity (I = 87.02%). 

On chromosome 7, the BR-QTL7 was identified at approx. 319 Mbp (Figure 5d). Linked to that QTL a gene, *evm.TU.7.2707* showed a higher association (−log_10_(*p*-value) = 4.10) to BRR. BLAST-N showed homology (I = 91.76%) with a cytochrome P450 (*CYP450*) from *A. tauschii,* while GO analysis unveiled that oxidation-reduction was its biological process.

More relevant genes that might play a role in the defense were identified when considering a −log_10_(*p*-value) > 4.0 in the MAGMA analysis (Table 5). BLAST-N information of the candidate genes identified by GCTA and MAGMA are listed in Table S6.

## 4. Discussion

In this investigation, phenotypic data from a field trial conducted during summer 2018 indicated that both infections caused by crown and brown rust had a severe impact on the tested ryegrass population (Figure S5). The correlation between the two traits was low, suggesting that the early brown rust infection did not affect the genotypes’ response to the subsequent crown rust infection. Moreover, none of the identified genes related to crown and brown rust response were shared between the two traits in the present study, suggesting the two diseases are regulated by different genes.

Next-generation sequencing technologies have enabled genomes to be resequenced at a significantly lower cost than ever before [36] and to generate high-density marker sets. In this study, a total of 14,538,978 SPNs were detected with an average of one SNP every 171 bp. The lack of clear stratification, as well as the presence of many rare alleles in the population, are due to the use of an unselected material of an outbreeding and highly heterozygous species. However, the high-density SNP dataset allowed us to estimate LD decay with the highest possible precision to date for perennial ryegrass. As expected, results showed a minimal LD extension with the rapid decay of *r*^2^ value below 0.2 after 520 bp on average.

Interestingly, LD decay for chromosome 7 was almost double of what was found for chromosome 4, indicating that the genetic variability greatly differs between chromosomes. An explanation for this may lie in the population development history of the genotypes. Although unselected and open-pollinated, there may still be genomic regions undergoing stronger selection than the average. For example, chromosomes harboring loci that affect flowering time or pollen production may account for a level of assortative mating. When comparing our results to Fè et al. [16], who used breeding material with some degree of inbreeding and reported an average distance marker having LD > 0.25 of 1200 bp, we could observe a faster LD decay in the unselected plant material included in our study. Although populations’ characteristics and methods to estimate LD decay may return different outputs, the differences between the results presented here and in Fè et al. [16] are striking. We speculated that genotyping by sequencing (GBS) derived SNPs may not be, for the largest part, physically close enough to precisely evaluate LD when decreasing so rapidly below *r*^2^ values of 0.2. At the same time, when considering decay below 0.1, the two studies returned comparable results of approximately 6–7 Kbp. Such rapid LD decay will have to be considered for future studies aiming at capturing whole genomic effects for marker-trait association and predictions. Even if GBS as a genotyping method for perennial ryegrass is efficient for genomic prediction, the GBS-based genotyping strategy may not be sufficiently dense throughout the whole genome to effectively capture all the genetic variance and all the causal variants linked to relevant phenotypic traits. 

The absence of a population structure, as well as the relatively low number of genotypes compared to the marker dataset dimension, are the main limitations that most likely caused the lack of statistically significant associations of SNPs under Bonferroni and false discovery rate (FDR) corrections when using GCTA. Results from a previous study [37] suggested the employment of a smaller sample size when testing common SNPs with a strong effect and a high LD (*r*^2^ > 0.4) between marker allele and disease allele. In contrast, our study showed how intra-chromosomal LD rapidly decay below 0.2 after a few hundred base pairs. Moreover, Hong et al. [37] suggested that studies with many SNP markers (1 million) require a large number of samples (1000 case-control) to achieve adequate statistical power. In addition, Wang et al. [38] proposed that a large population size may be necessary for GWAS when analyzing quantitative traits, such as disease resistance. Therefore, it is likely that significant associations went undetected due to this lack of statistical power.

However, this study presents for the first time a marker-trait association study on perennial ryegrass based on resequencing, which will pave the way for future studies as the sequencing costs continue to decrease.

### 4.1. Candidate Genes for Crown Rust Resistance

Based on BLAST-N analysis, none of the identified genes found similarities to known rust-related genes in other species. Nevertheless, the information collected by their biological processes, molecular functions, and sequence homology with already annotated genes is in line with our expectation since they cover essential functions in the plant-pathogen interaction. The MAGMA approach identified one gene with a statistically significant association to crown rust resistance on chromosome 6 (*evm.TU.6.9029*) that we called *LpPc6* (the initials of the plant and pathogen, followed by the chromosomal number position), and more genes were then detected with a −log_10_(*p*-value) > 4.0. This result suggests that the markers’ association with the trait may change when the SNPs are aggregated to the gene level compared with GWAS using GCTA. The most important reason for that is the reduction in the number of features from more than 14 million SNPs (GCTA) to approximately 49,000 genes (MAGMA) which increases the statistical power. 

Our findings are consistent with prior research on plant-pathogen interactions [39,40]. Indeed, interconnecting signaling pathways regulate perennial ryegrass’s defense response against pathogens such as crown rust. The primary component is recognizing pathogen elicitors by the plant, which triggers the host signal transduction pathway. A crown rust spore sends out its germ tube seeking stomata pores on the outer epidermal cell of the plant. A wax surface covers these cell walls [41], mainly made of triterpenes, which might interact with the pathogen acting as signal molecules. In our study, the gene *evm.TU.3.855* showed homology with a gene involved in the first steps of the avenacin pathway, which are antifungal triterpenes that in oats are known to provide protection against soilborne diseases [34]. 

Once inside the plant, infection hyphae grow to colonize the intercellular space [10]. At this stage, TMK proteins, the most well-known R proteins, might activate kinase enzymes following recognition of pathogen elicitors [42]. The gene *evm.TU.2.2845* found homology with a *TMK*-gene suggesting that it might play a role in recognizing specific elicitors released by the pathogen and therefore, activate a cytoplasmatic kinase involved in signal transduction. Two previous studies [43,44] revealed two QTLs located on LG2 (*LpPc3* and *LpPc1*) involved in crown rust resistance in perennial ryegrass. Our results reported a QTL on the same chromosome, CR-QTL-2.1, which may be referred to as *LpPc3*, at approx. 208 Mbp, and a second QTL, CR-QTL-2.2 at approx. 343 Mbp close to the before-mentioned *evm.TU.2.2845* gene. We speculate that CR-QTL-2.2 refers to *LpPc1*; however, it was not possible to verify it given the information available from the cited studies. 

Once the fungus is inside the cell’s cytoplasm, protein kinases may function as signaling receptors, which might be activated to induce a cascade of signaling to activate the defense response. The statistically significant gene associated with crown rust resistance, *LpPc6*, had high homology to a *CK2* belonging to the serine-threonine kinase family, which phosphorylate numerous substrates. Rödiger et al. [45] reported the CK2’s involvement in photosynthetic regulation indicating its function as a balancer between various metabolic activities. Photosynthesis is inhibited by pathogens such as crown rust, which damages leaf tissue, revealing that CK2 may be implicated in the resistance. Previous research on several plant-rust interactions found that the photosynthetic pathway was one of the most altered in response to pathogen infection [46,47,48]. Furthermore, knocking down the *CK2* gene in *A. thaliana* increased stomatal aperture and leaf water loss [49]. Because the pathogen enters the plant through the stomatal pore, *LpPc6* might be involved in the early response to crown rust infection. How this is accomplished and regulated remained unclear; however, protein kinases play an important and well-known role in signal transduction, culminating in hypersensitive responses. Other candidate genes implicated in elicitor detection inside the cytoplasm include *evm.TU.1.12851*, which codes for a wall-associated receptor kinase (WAK), and *evm.TU.7.24966*, which is homologous to an *NBS-LRR* resistance gene. WAKs role in pathogen resistance has already been described [50], and previous studies identified two loci related to crown rust resistance on chromosome 1 [43,44], suggesting that the candidate *WAK* gene might be linked to the already known QTL. On the other hand, BLAST-N analysis revealed homology between the *NBS-LRR* resistant gene with an *R* gene (*Pik2*) located at the Pik locus, which in rice is involved in the resistance to many rice blast isolates (*Magnaporthe grisea*) [51]. Among the race-specific rust resistance genes, the ones containing the *NBS-LRR* domains are best characterized in wheat, giving resistance to stripe-, leaf-, and stem rust [47,52,53].

Overall, defense response genes need to be activated as a response to pathogen infection. In plants, response to pathogens is regulated by signaling pathways in which the primary components are signal molecules like salicylic acid (SA) and jasmonic acid (JA), which accumulate upon pathogen infection to trigger defense mechanisms [42,54]. The candidate gene *evm.TU.3.8552*, which is homologous to a *VQ* motif gene, may play a role in SA and JA-mediated plant response by interacting with various transcription factors and triggering the expression of defense-responsive genes [33]. Furthermore, *evm.TU.4.2271*, which had a strong association with crown rust resistance and is adjacent to the CR-QTL-4, shared similarities with a gene encoding a protein from the NRT1/PTR family (NPF). According to recent research, NPFs are involved in transporting plant hormones and secondary metabolites, and these genes are substantially activated by biotic and abiotic treatments [55,56]. Crown rust resistance QTL have previously been identified on chromosome 4 [57], but due to differences in the investigated populations, we could not determine whether the previously annotated loci were the same as those reported in our study. 

In addition to that, a recent study detected five markers associated with crown rust in oats, where homology with proteins related to the plant immune defense reaction was identified [58]. However, there was no similarity with those identified in our study, indicating that although analogous pathways might be involved in crown rust infection response, there are also differences. Moreover, in sorghum (*Sorghum bicolor*), multiple QTL were discovered over all the ten chromosomes [59], confirming the resistance’s complexity and polygenic nature and suggesting that in perennial species, such as *L. perenne*, a more durable resistance is preferred due to the longer life cycle. Future researches are required to validate the findings of this work. Bulk segregant analysis [60] for example, might be an alternate method for identifying molecular markers associated with these target genes or the found QTLs in two bulks with distinct phenotypic differences.

### 4.2. Candidate Genes for Brown Rust Resistance

Regarding brown rust resistance, no statistically significant SNPs were identified through GCTA, but over the arbitrary threshold, 47 SNPs were detected equally distributed across the seven chromosomes, and 27 genes were annotated within a region of 5 Kbp. One gene on chromosome 6, *evm.TU.15799*, which we called *LpPl6*, showed a statistically significant association with brown rust resistance by the MAGMA approach, and seven other genes were found with a strong association to the trait. This is the first time that genes involved in response to brown rust caused by *P. loliina* have been identified. 

Genetic resistance against brown rust may involve similar mechanisms as other plant-pathogen interactions. Some plant plasma membrane (PM) proteins target specific pathogen’s extrahaustorial membrane proteins to activate a defense response, such as the LRR proteins Cf-2,-4,-5,-9 in tomatoes, giving resistance to *C. fulvum* [61], and the broad-spectrum protein RPW8 in *A. thaliana* [62]. Genes encoding PM proteins were identified through both the GCTA and MAGMA approaches in the current study. The *LpPl6* gene shared a significant degree of similarity with the *IP5PTase*-like gene, which possesses phosphatase activity against cell membrane receptors such as Ins(1,4,5)P3 [63], detecting and amplifying signals from outside the cell. A study in *A. thaliana* reported that a mutation in the gene reduced ROS production and decreased the expression of stress-responsive genes in abiotic stress conditions [64]. We hypothesize a similar role for *LpPl6* for brown rust resistance; however, how this is regulated remains a topic for further research. Another PM protein might be expressed by the *evm.TU.1.1542* gene, which showed similarity to a *WAK* encoding gene. Evidence that WAKs play a role in pathogen resistance has been reported both in the model plant Arabidopsis against *Fusarium* [65] and in crops such as rice against rice blast disease [66]. Another gene encoding for a PM protein, *evm.TU.7.22707*, had homology to a gene encoding a membrane-associated protein of the CYP450 superfamily. The role of CYP450 in plant defense is linked to the phytoalexin biosynthesis, hormone metabolism, and control of secondary metabolite biosynthesis [67], triggering the activation of defense response mechanisms. 

Pathogen elicitors may interact with cytoplasmatic proteins, activating many cellular pathways leading to the defense response. A gene, *evm.TU.2.3895*, found a similarity to an *NCS*, which is classified as a member of the Pathogen-Related 10/Bet v1 Protein Family (PR10). Despite the unclear role of PR10 proteins in plant defense, it has been reported to be involved in Arabidopsis defense response to *F. oxysporum*, *B. cinerea*, and *P. syringe* [68], in maize against *Aspergillus flavus* [69], and to *Alternaria solani* in tobacco [70].

Recognition of the pathogen-produced signal molecules activates a cascade of plant biochemical reactions, including a rapid burst of reactive oxygen species where peroxidases play an essential role in catalyzing the oxidative reaction involving H_2_O_2_ [71]. Two genes, *evm.TU.7.19634* and *evm.TU.719642*, were detected with similarity to a peroxidase (*P7*)-like gene. These findings suggest that elicitor recognition might occur during the plant-pathogen interaction, resulting in the activation of the cytoplasmic peroxidases for potential race-specific resistance.

Following recognition, signal transduction regulates gene expression that may directly or indirectly play an essential role in stopping the infection. Several genes encoding for stress-responsive genes such as F-box/LRR repeat protein (*evm.TU.5.11861* and *evm.TU.5.11862*), a glycine-rich cell wall structure protein (*evm.TU.7.6143*), and a TIFY protein (*evm.TU.7.11390*) were identified. In several studies, the expression of the above-mentioned genes changed under biotic stress, suggesting that they were important in activating defense mechanisms against pathogens [72,73,74].

Furthermore, regulation of gene expression and RNA processing, such as splicing of messenger RNA precursor, is essential during signal transduction to produce proteins that may activate a successful defense response. The gene *evm.TU.2.3894* found homology to a gene encoding for a serine/arginine-rich (SR) protein important for spliceosome assembly and cell survival [75]. A previous study reported the effect of hormones and temperature stress on the SR splicing activity [76]. No research has been focused on the influence of biotic stresses, such as brown rust attack, on the SR genes’ alternative splicing. However, SR genes might likely undergo alternative splicing to produce structurally and functionally different proteins under biotic stress conditions. 

Plants have evolved different mechanisms to adapt to unfavorable conditions, and many of these involved plant hormone signaling pathways, where ethylene (ET) plays an essential role in inducing biotic stress response defense [77,78,79]. A gene, the *evm.TU.2.23247*, had high homology to a gene that encodes for a transcription factor AP2 ethylene-responsive factor (Ape/ERF), which emerged as essential during stress conditions responding to ET to help activate ET stress-responsive genes [80].

Our results present for the first time the interaction between perennial ryegrass and the fungus *P. loliina* at the molecular level. The identified QTL regions and candidate genes could be a potential new source of resistance in perennial ryegrass but should be further investigated to better understand and breed for brown rust tolerance.

## 5. Conclusions

The resequencing of a diploid perennial ryegrass population phenotyped for crown and brown rust resistance produced a large and complex dataset employed in a genome-wide association study. The dataset represented a set of markers physically close enough to evaluate LD more precisely than GBS-derived SNPs when testing a population of an outbreeding species with a rapid LD decay. Among all the methods to perform GWAS, some, such as the GCTA and MAGMA approaches, are more suited to perform the analysis of a large dataset, significantly reducing the running time and memory required. The relatively low number of genotypes included in the study compared to the marker dataset dimension may explain the lack of statistically significant associations after Bonferroni and FDR corrections using GCTA. However, when a less stringent threshold was selected, markers for both traits were detected. Genes within 5 Kbp from the most significant SNPs were identified, showing homology to genes involved in response to pathogen infection. On the other hand, the MAGMA approach identified one gene statistically significant associated with crown rust resistance on chromosome 6 (*LpPc6*) and another gene on the same chromosome with brown rust resistance (*LpPl6*). Based on these findings, we can hypothesize that the association of the markers with the trait may vary when the SNPs are aggregated to the gene level. Both *LpPc6* and *LpPl6* encode proteins with phosphatase activity, which we hypothesize can be induced by the host to perceive, amplify, and then transfer signals to downstream components, thus activating a plant defense response. Those genes might be of particular interest providing candidate genes for future studies validating their role through knockout experiments or by testing their gene expression profile following disease infection.

## Figures and Tables

**Table 1:** Summary statistics and trait repeatability estimates for BR and CR. Min refers to the lowest scored value; Max refers to the highest scored value; SD refers to the standard deviation, Cvar refers to the coefficient of variation.

Traits	BR	CR
Min	2	0
Max	9	9
Mean	6	4
Variance	3.09	4.21
SD	1.76	2.05
Cvar (%)	29	48
Repeatability	0.78	0.70

**Table 2:** Summary of SNPs distribution. Perennial ryegrass chromosome size (Chr size (bp)), number of SNPs identified per chromosome (SNPs/Chr), and the average SNP density (SNP/bp). A total of 259,221 SNPs were located on scaffolds.

Chromosome	Chr Size (bp)	SNPs/Chr	SNP/bp
Chr1	271,335,344	1,781,903	152
Chr2	346,255,425	2,254,571	154
Chr3	383,839,144	2,224,970	173
Chr4	414,259,934	2,753,346	150
Chr5	259,831,545	1,545,724	168
Chr6	276,808,772	1,774,834	156
Chr7	359,413,018	1,944,409	185

**Table 3:** Summary results of GCTA. Additive (A), phenotypic (P), residuals (e) variance (V) and SNP heritability (*h^2^_SNP_*) explained for crown rust (CR) and brown rust (BR) resistance. Numbers between brackets refer to standard deviation.

Variables	V(A)	V(P)	V(e)	*h^2^*_SNP_
CR	2.08 (1.82)	4.97 (0.46)	2.82 (1.75)	0.42 (0.35)
BR	2.88 (1.42)	3.56 (0.33)	0.68 (1.31)	0.81 (0.37)

**Table 4:** Genes associated with CRR identified using MAGMA (−log_10_(*p*-value) > 4.0). Chromosome number (Chr); Gene coordinate (bp) on the chromosome (Start and Stop); numbers of SNPs detected within the gene sequence (N SNP); *p*-value for the association of the genes with the CRR (*p*-value).

Gene ID	Chr	Start	Stop	N SNPs	−log10(*p*-Value)
evm.TU.2.24844	2	343,028,981	343,029,284	3	5.72
evm.TU.3.8555	3	123,435,108	123,443,087	31	4.63
evm.TU.3.20827	3	295,634,059	295,635,654	25	5.22
evm.TU.4.2271	4	26,600,949	26,602,802	58	4.65
evm.TU.6.9029	6	126,251,598	126,256,062	48	6.39
evm.TU.7.24966	7	350,687,605	350,692,741	143	4.58

**Table 5:** Genes associated with BRR identified using MAGMA (−log_10_(*p*-value) > 4.0). Chromosome number (Chr); Gene coordinate (bp) on the chromosome (Start and Stop); numbers of SNPs within the gene (N SNP); *p*-value for testing the association of the genes with BRR (*p*-value).

Gene ID	Chr	Start	Stop	N SNPs	−log_10_(*p*-Value)
evm.TU.2.3894	2	52,178,587	52,182,215	131	4.39
evm.TU.2.19756	2	277,159,670	277,161,603	34	5.12
evm.TU.3.8432	3	121,925,172	121,925,559	1	4.43
evm.TU.3.8507	3	122,870,603	122,872,466	-	4.47
evm.TU.3.8509	3	122,875,455	122,903,941	34	4.93
evm.TU.3.9565	3	138,177,359	138,179,818	14	4.86
evm.TU.3.23458	3	332,049,981	332,054,690	-	4.17
evm.TU.3.25826	3	363,848,840	363,852,037	60	4.74
evm.TU.5.11920	5	164,892,059	164,894,440	-	4.22
evm.TU.6.3022	6	37,956,882	37,960,523	58	4.50
evm.TU.6.15799	6	219,584,326	219,586,833	56	6.22
evm.TU.6.15800	6	219,587,444	219,589,409	55	4.46
evm.TU.7.22707	7	319,181,319	319,183,513	59	4.10

## Supplementary Materials

The following are available online at https://www.mdpi.com/article/10.3390/genes13010020/s1, Figure S1: Flowchart of population development, Figure S2: Phenotypic distribution among the 1,000 genotypes of crown (a) and brown rust (b), Figure S3: Site Frequency Spectrum (SFS) plot, Figure S4: Manhattan plot GWAS results of CRR (a) and BRR (b), Table S1: List of SNPs associated with CR, Table S2: List of genes associated with CRR, Table S3: BLAST-N information of genes identified using GCTA and MAGMA for CRR, Table S4: List of SNPs associated with BRR, Table S5: List of genes associated with BRR, Table S6: BLAST-N information of genes identified using GCTA and MAGMA for BRR, Figure S5: Symptoms of crown rust infection on perennial ryegrass.
