# Supplementary material for: Genome-Wide Association Mapping of Crown and Brown Rust Resistance in Perennial Ryegrass
Source: Genes (Basel). 2021 Dec 22;13(1):20. doi: 10.3390/genes13010020 (PMC8774571; doi:10.3390/genes13010020)
Supplement: Supplementary file 1 [file genes-13-00020-s001.zip › genes-1513847-supplementary.pdf]

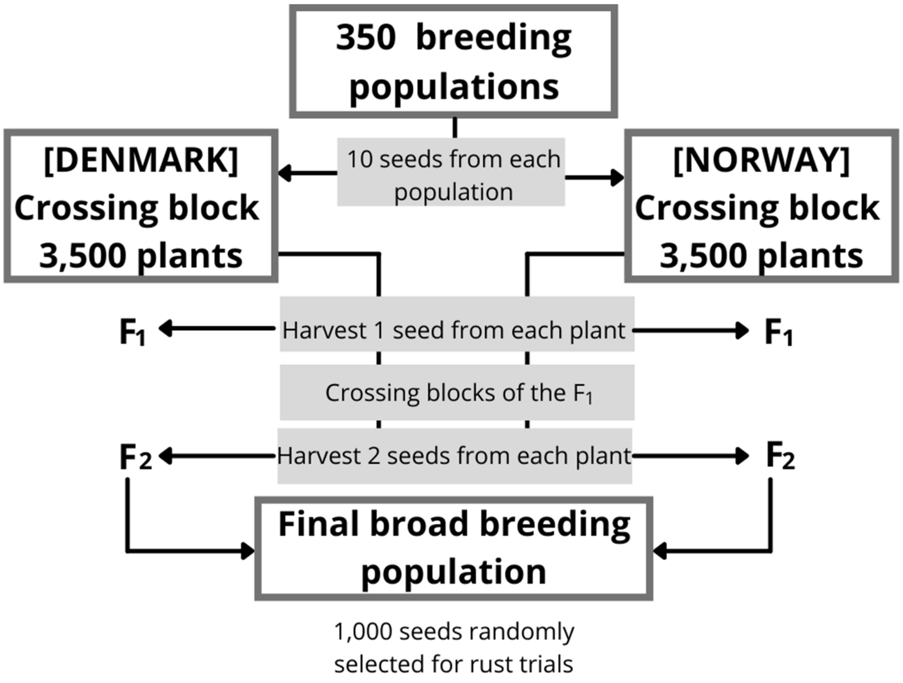

Figure S1. Flowchart of population development.

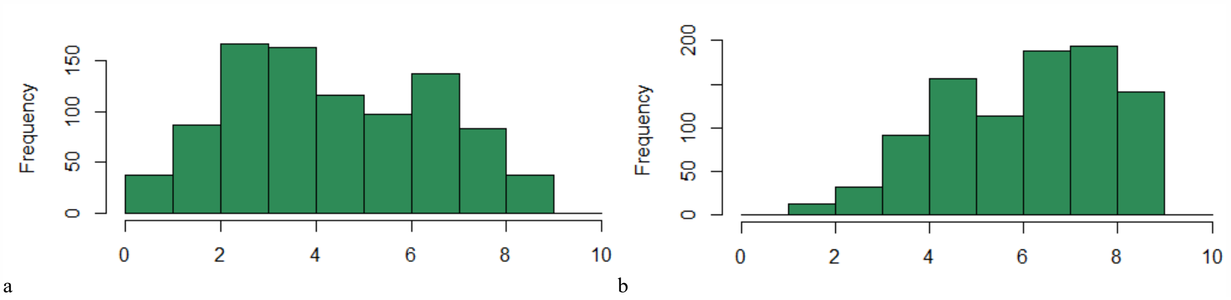

Figure S2. Phenotypic distribution among the 1,000 genotypes of crown (a) and brown rust (b). The x-axis refers to the scale used for the disease scoring, where 1 refers to heavy attack (high susceptibility of perennial ryegrass) and 9 corresponds to no rust infection (high tolerance).

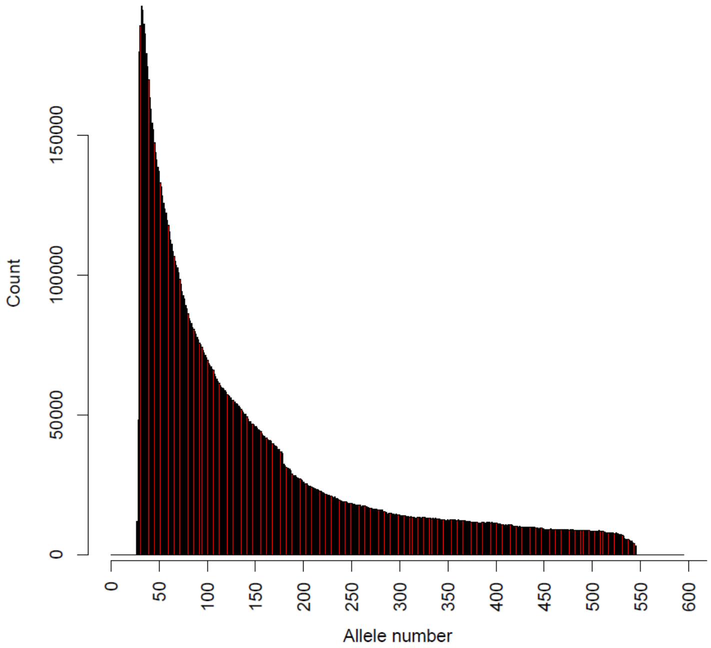

Figure S3. Site Frequency Spectrum (SFS) plot. Distribution of allele frequencies of the SNP loci in the population

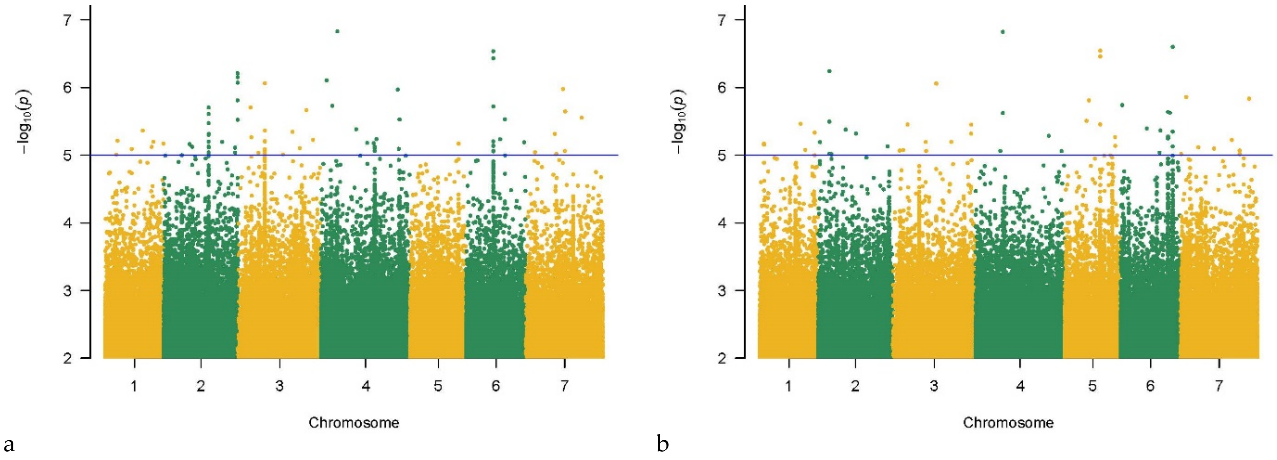

Figure S4. Manhattan plot GWAS results of CRR (a) and BRR (b). On the x-axis, the 7 chromosomes are reported in alternating colours. The blue horizontal line indicates the arbitrary cutoff of $-\log_{10}(p\text{-value})=5$.

Table S1. List of SNPs associated with CR. Selected markers with a *p*-value above the arbitrary threshold $-\log_{10}(p\text{-value})>5$.

SNPs ID	Chr	Position (bp)	p-value
**Gcta-snp-1.1**	1	50,888,681	8.82976e-06
**Gcta-snp-1.2**	1	57,202,120	6.85289e-06
**Gcta-snp-1.3**	1	123,289,042	7.1705e-06
**Gcta-snp-1.4**	1	17,455,1781	4.39621e-06
**Gcta-snp-1.5**	1	216,254,732	7.8503e-06

**Gcta-snp-1.6**	1	223,812,092	6.54172e-06
**Gcta-snp-1.7**	1	270,501,016	7.68761e-06
**Gcta-snp-2.1**	2	84,442,240	9.82199e-06
**Gcta-snp-2.2**	2	120,467,462	7.239e-06
**Gcta-snp-2.3**	2	132,102,685	8.4921e-06
**Gcta-snp-2.4**	2	208,763,776	1.8167e-06
**Gcta-snp-2.5**	2	208,763,879	6.0652e-06
**Gcta-snp-2.6**	2	208,765,282	8.73605e-06
**Gcta-snp-2.7**	2	208,806,413	9.51495e-06
**Gcta-snp-2.8**	2	208,810,723	9.9128e-06
**Gcta-snp-2.9**	2	208,811,051	3.20473e-06
**Gcta-snp-2.10**	2	208,829,396	8.9686e-06
**Gcta-snp-2.11**	2	208,834,020	2.23917e-06
**Gcta-snp-2.12**	2	208,834,440	4.63526e-06
**Gcta-snp-2.13**	2	208,848,996	7.28031e-06
**Gcta-snp-2.14**	2	209,769,507	4.5522e-06
**Gcta-snp-2.15**	2	271,678,020	6.66405e-06
**Gcta-snp-2.16**	2	331,778,177	9.69674e-06
**Gcta-snp-2.17**	2	331,831,873	8.31896e-06
**Gcta-snp-2.18**	2	343,028,606	8.33522e-07
**Gcta-snp-2.19**	2	343,028,650	6.93544e-07
**Gcta-snp-2.20**	2	343,029,116	5.74696e-07
**Gcta-snp-3.1**	3	56,959,112	1.83089e-06
**Gcta-snp-3.2**	3	123,400,748	7.75118e-07
**Gcta-snp-3.3**	3	123,493,806	4.20124e-06
**Gcta-snp-3.4**	3	123,499,279	9.91972e-06
**Gcta-snp-3.5**	3	123,511,283	5.67409e-06
**Gcta-snp-3.6**	3	251,948,944	4.38419e-06
**Gcta-snp-3.7**	3	285,814,253	7.77014e-06
**Gcta-snp-3.8**	3	316,837,904	2.28292e-06
**Gcta-snp-3.9**	3	347,482,523	6.66388e-06
**Gcta-snp-4.1**	4	26,602,446	8.24678e-07
**Gcta-snp-4.2**	4	53,000,213	2.20674e-06
**Gcta-snp-4.3**	4	76,349,694	1.57119e-07
**Gcta-snp-4.4**	4	163,621,753	3.93225e-06
**Gcta-snp-4.5**	4	214,582,294	6.8227e-06
**Gcta-snp-4.6**	4	244,680,870	7.4836e-06
**Gcta-snp-4.7**	4	249,794,945	4.76004e-06
**Gcta-snp-4.8**	4	257,511,725	5.6311e-06
**Gcta-snp-4.9**	4	356,839,682	1.14406e-06
**Gcta-snp-4.10**	4	361,318,775	8.68191e-06
**Gcta-snp-4.11**	4	365,157,378	3.22589e-06
**Gcta-snp-5.1**	5	224,633,296	6.94505e-06
**Gcta-snp-6.1**	6	126,252,299	8.77432e-06
**Gcta-snp-6.2**	6	126,253,264	7.34757e-06
**Gcta-snp-6.3**	6	126,253,475	6.38375e-06
**Gcta-snp-6.4**	6	126,254,672	3.6641e-07

**Gcta-snp-6.5**	6	126,255,271	4.86194e-06
**Gcta-snp-6.6**	6	158,207,491	6.63217e-06
**Gcta-snp-6.7**	6	270,303,728	5.81906e-06
**Gcta-snp-7.1**	7	43,143,239	9.11627e-06
**Gcta-snp-7.2**	7	172,435,701	1.07606e-06
**Gcta-snp-7.3**	7	181,138,453	9.07996e-06
**Gcta-snp-7.4**	7	183,420,642	2.32578e-06
**Gcta-snp-7.5**	7	259,535,462	2.9603e-06

Table S2. List of genes associated with CRR. Gene ID refers to the name of the gene, Chr refers to the chromosome where the gene is located, START and STOP refer to the gene's first and last nucleotide position on the chromosome, and Gene size refers to the gene size in bp.

Gene ID	Chr	START	STOP	Gene size (bp)
**Lp_chr1_0.12850**	chr1	174,544,889	174,548,709	3820
**Lp_chr1_0.12851**	chr1	174,550,364	174,551,550	1186
**Lp_chr1_0.16593**	chr1	223,810,306	223,812,121	1815
**Lp_chr2_0.6271**	chr2	84,445,123	84,448,803	3680
**Lp_chr2_0.8753**	chr2	120,466,171	120,468,611	2440
**Lp_chr2_0.24844**	chr2	343,028,981	343,029,284	303
**Lp_chr2_0.24845**	chr2	343,029,675	343,033,480	3805
**Lp_chr3_0.8551**	chr3	123,396,008	123,396,638	630
**Lp_chr3_0.8552**	chr3	123,400,198	123,400,828	630
**Lp_chr3_0.17611**	chr3	251,942,496	251,944,253	1757
**Lp_chr3_0.20045**	chr3	285,813,172	285,815,144	1972
**Lp_chr3_0.24613**	chr3	347,481,574	347,482,756	1182
**Lp_chr3_0.24615**	chr3	347,485,871	347,488,371	2500
**Lp_chr4_0.2271**	chr4	26,600,949	26,602,802	1853
**Lp_chr4_0.6124**	chr4	76,351,602	76,351,911	309
**Lp_chr4_0.6125**	chr4	76,353,862	76,356,228	2366
**Lp_chr4_0.18309**	chr4	244,684,987	244,688,942	3955
**Lp_chr4_0.19179**	chr4	257,503,274	257,507,594	4320
**Lp_chr4_0.19180**	chr4	257508,178	257,509,165	987
**Lp_chr4_0.19181**	chr4	257,513,104	257,514,895	1791
**Lp_chr6_0.9029**	chr6	126,251,598	126,256,062	4464
**Lp_chr7_0.1.18459**	chr7	259,529,572	259,531,605	2033

Table S3. BLAST-N information of genes identified using GCTA and MAGMA for CRR. Expected Value (E-value) refers to the number of expected hits of similar quality that could found just by chance with the calculated score. Coverage (Cov) refers to the percent of the query length included in the aligned segments. Identity (I) refers to the highest percent identity for a set of aligned segments to the same subject sequence.

Gene ID	Organism	E-value	Cov (%)	I (%)	Accession number	Putative function
**Lp_chr1_0.12850 (GCTA)**	*A. tauschii*	0	100	90.53	XM_020337866.1	protein root UVB sensitive 6-like

**Lp_chr1_0.12851 (GCTA)**	*A. tauschii*	3.00e-112	66	80.22	XM_020323781.1	wall-associated receptor kinase
**Lp_chr1_0.16593 (GCTA)**	*B. distachyon*	0	99	87.55	XM_003568324.4	tubby-like protein
**Lp_chr2_0.6271 (GCTA)**	*A. tauschii*	0	99	93.76	XM_020335834.1	cinnamate 4-monooxygenase-like
**Lp_chr2_0.22569 (MAGMA)**	-	-	-	-	-	
**Lp_chr2_0.24844 (GCTA, MAGMA)**	-	-	-	-	-	
**Lp_chr2_0.24845 (GCTA)**	*A. tauschii*	0	47	86.75	XM_020322177.1	a transmembrane receptor protein kinase TMK1
**Lp_chr2_0.8753 (GCTA, MAGMA)**	*B. distachyon*	3.00e-04	2	97.14	XM_024461354.1	
**Lp_chr3_0.8552 (GCTA)**	*A. tauschii*	0	100	91.08	XM_020298474.1	VQ motif
**Lp_chr3_0.8555 (GCTA-MAGMA)**	*Avena strigosa*	0	62	84.90	DQ680849.1½	Sad1 and Sad2
**Lp_chr3_0.17805 (GCTA, MAGMA)**	*A. tauschii*	0	96	80	XM_020296375.1	oxygenase called indole-3-pyruvate monooxygenase (YUCCA10)
**Lp_chr3_0.20704 (GCTA)**	*L. perenne*	0	97	94.15	KY771172.1	beta-1-6-glucanase-like protein
**Lp_chr3_0.20827 (MAGMA)**	*A. tauschii*	0	91	81.12	XM_020342790.1	4hydroxyphenylacetaldehyde oxime monooxygenase-like
**Lp_chr4_0.2271 (GCTA, MAGMA)**	*A. tauschii*	0	96	87.01	XM_020293186.1	NRT1/PTR family 5.7-like
**Lp_chr4_0.6084 (GCTA)**	*A. tauschii*	0	64	86.05	XM_003560396.4	phosphatidylinositol transfer
**Lp_chr4_0.6125 (GCTA)**	*A. tauschii*	0	91	77.39	XM_020318203.1	chaperone protein ClpB1-like.
**Lp_chr4_0.18630 (GCTA, MAGMA)**	*A. tauschii*	0	99	87.81	XM_020306365.1	metacaspase-1-like.
**Lp_chr4_0.18634 (GCTA, MAGMA)**	*A. tauschii*	0	100	84.44	XM_020298243.1	metacaspase-1-like.
**Lp_chr4_0.19180 (GCTA)**	*A. tauschii*	0	100	87.78	XM_020343272.1	pentatricopeptide repeat (PRR) proteins
**Lp_chr4_0.20313 (GCTA)**	*A. tauschii*	0	66	86.02	XM_020296375.1	oxygenase called indole-3-pyruvate monooxygenase (YUCCA10)

**Lp_chr5_0.16081 (MAGMA)**	*A. tauschii*	2.00e-33	8	82.97	KY771172.1	beta-1-6-glucanase-like protein.
**Lp_chr6_0.9029 (GCTA, MAGMA)**	*A. tauschii*	0	100	93.39	XM_020321879.1	plastid casein kinase 2 (CK2)
**Lp_chr7_0.1.9979 (GCTA)**	*L. perenne*	6.00e-07	2	100	KY771172.1	beta-1-6-glucanase-like protein.
**Lp_chr7_0.1.11324 (GCTA)**	*B. distachyon*	0	100	86.13	XM_003574460.4	ubiquitin receptor RAD23.
**Lp_chr7_0.1.24966 (MAGMA)**	*Triticum dicoccoides*	0	96	82.60	XM_037607392.1	Disease resistance protein Pik-2.

Table S4. List of SNPs associated with BRR. Selected markers with a *p*-value above the arbitrary threshold $-\log_{10}(p\text{-value})>5$.

SNP ID	Chr	position	p-value
**BR-Gcta-snp-1.1**	1	20,121,295	6.98e-01
**BR-Gcta-snp-1.2**	1	20,121,300	6.57e-01
**BR-Gcta-snp-1.3**	1	189,974,240	3.56e-01
**BR-Gcta-snp-1.4**	1	211,929,165	9.57e-01
**BR-Gcta-snp-1.5**	1	255,276,992	4.56e-01
**BR-Gcta-snp-2.1**	2	8,243,648	6.30e-01
**BR-Gcta-snp-2.2**	2	52,157,077	3.17e-01
**BR-Gcta-snp-2.3**	2	52,180,401	9.51e-01
**BR-Gcta-snp-2.4**	2	52,180,410	6.12e-02
**BR-Gcta-snp-2.5**	2	61,858,721	9.92e-01
**BR-Gcta-snp-2.6**	2	127,577,619	4.62e-01
**BR-Gcta-snp-2.7**	2	175,999,676	4.94e-01
**BR-Gcta-snp-2.8**	2	323,147,284	8.09e-01
**BR-Gcta-snp-3.1**	3	32,254,304	8.91e-01
**BR-Gcta-snp-3.2**	3	48,819,030	7.89e-01
**BR-Gcta-snp-3.3**	3	68,544,650	3.42e-01
**BR-Gcta-snp-3.4**	3	152,867,482	6.66e-02
**BR-Gcta-snp-3.5**	3	154,994,124	9.03e-01
**BR-Gcta-snp-3.6**	3	201,638,596	8.82e-02
**BR-Gcta-snp-3.7**	3	271,821,353	6.63e-01
**BR-Gcta-snp-3.8**	3	363,850,690	5.40e-01
**BR-Gcta-snp-4.1**	4	116,156,855	8.11e-01
**BR-Gcta-snp-4.2**	4	127,117,474	2.39e-02
**BR-Gcta-snp-4.3**	4	127,117,513	1.44e-02
**BR-Gcta-snp-4.4**	4	339,683,441	5.37e-01
**BR-Gcta-snp-4.5**	4	399,341,149	8.16e-01
**BR-Gcta-snp-5.1**	5	101,345,714	3.42e-01
**BR-Gcta-snp-5.2**	5	112,656,442	1.69e-01
**BR-Gcta-snp-5.3**	5	164,117,281	3.77e-01
**BR-Gcta-snp-5.4**	5	164,899,699	3.58e-02

**BR-Gcta-snp-5.5**	5	233,960,573	5.79e-01
**BR-Gcta-snp-5.6**	5	234,361,710	7.44e-01
**BR-Gcta-snp-6.1**	6	7,336,647	1.86e-01
**BR-Gcta-snp-6.2**	6	121,008,266	4.23e-01
**BR-Gcta-snp-6.3**	6	180,310,095	9.58e-01
**BR-Gcta-snp-6.4**	6	184,483,550	4.34e-01
**BR-Gcta-snp-6.5**	6	219,585,587	2.51e-01
**BR-Gcta-snp-6.6**	6	219,586,506	5.76e-01
**BR-Gcta-snp-6.7**	6	227,975,953	2.51e-01
**BR-Gcta-snp-6.8**	6	241,419,154	7.54e-01
**BR-Gcta-snp-7.1**	7	27,365,296	1.38e-01
**BR-Gcta-snp-7.2**	7	83,124,103	7.53e-01
**BR-Gcta-snp-7.3**	7	156,537,933	8.19e-01
**BR-Gcta-snp-7.4**	7	238,806,220	6.23e-01
**BR-Gcta-snp-7.5**	7	275,454,738	9.88e-01
**BR-Gcta-snp-7.6**	7	27,5494,480	8.77e-01
**BR-Gcta-snp-7.7**	7	319,181,966	1.44e-01

Table S5. List of genes associated with BRR. Gene ID refers to the name of the gene Chr refers to the chromosome where the gene is located, START and STOP refer to the gene's first and last nucleotide position on the chromosome, and Gene size refers to the gene size in bp.

Gene ID	Chr	START	STOP	Gene size (bp)
**Lp_chr1_0.1542**	chr1	20,120,371	20,121,325	954
**Lp_chr1_0.18994**	chr1	255,276,205	255,278,383	2,178
**Lp_chr1_0.18995**	chr1	255,281,635	255,290,751	9,116
**Lp_chr2_0.622**	chr2	8,234,314	8,239,230	4,916
**Lp_chr2_0.623**	chr2	8,240,880	8,244,851	3,971
**Lp_chr2_0.3894**	chr2	52,178,587	52,182,215	3,628
**Lp_chr2_0.3895**	chr2	52,184,051	52,184,678	627
**Lp_chr2_0.23247**	chr2	323,143,203	323,146,714	3,511
**Lp_chr2_0.23248**	chr2	323,151,743	323,152,142	399
**Lp_chr3_0.3323**	chr3	48,815,776	48,816,132	356
**Lp_chr3_0.25825**	chr3	363,840,342	363,847,474	7,132
**Lp_chr3_0.25826**	chr3	363,848,840	363,852,037	3,197
**Lp_chr4_0.29466**	chr4	399,338,819	399,341,984	3,165
**Lp_chr4_0.29467**	chr4	399,343,955	399,344,255	300
**Lp_chr5_0.11861**	chr5	164,112,771	164,114,235	1,464
**Lp_chr5_0.11862**	chr5	164,117,453	164,119,064	1,611
**Lp_chr5_0.11921**	chr5	164,901,677	164,902,350	673
**Lp_chr5_0.16787**	chr5	234,358,978	234,359,638	660
**Lp_chr6_0.596**	chr6	7,338,230	7,341,436	3,206
**Lp_chr6_0.15799**	chr6	219,584,326	219,586,833	2,507
**Lp_chr6_0.15800**	chr6	219,587,444	219,589,409	1,965
**Lp_chr7_0.1.1922**	chr7	27,361,869	27,366,349	4,480
**Lp_chr7_0.1.6143**	chr7	83,126,037	83,126,433	396

**Lp_chr7_0.1.11390**	chr7	156,531,318	156,533,534	2,216
**Lp_chr7_0.1.19634**	chr7	275,454,123	275,455,236	1,113
**Lp_chr7_0.1.19642**	chr7	275,494,595	275,495,775	1,180
**Lp_chr7_0.1.22707**	chr7	319,181,319	319,183,513	2,194

Table S6. BLAST-N information of genes identified using GCTA and MAGMA for BRR. Expected Value (E-value) refers to the number of hits of similar quality that could be found just by chance. Coverage (Cov) refers to the percent of the query length included in the aligned segments. Identity (I) refers to the highest percent identity for a set of aligned segments to the same subject sequence.

Gene ID	Organism	E value	Cov (%)	I (%)	Accession number	Putatative function
**evm.model.Lp_chr1_0.1542 (GCTA)**	*A. tauschii*	8e-153	88	78.87	XM_020345349.1	wall-associated receptor kinase (WAK)
**evm.model.Lp_chr1_0.1598 (MAGMA)**	*A. tauschii*	0	94	82.97	XM_020341457.1	disease resistance protein RPM1-like
**evm.model.Lp_chr1_0.2823 (MAGMA)**	-	-	-	-	-	-
**evm.model.Lp_chr1_0.18994 (GCTA)**	*A. tauschii*	7e-157	48	77.05	XM_020327116.1	BPT/POZ and MATH domain-containing protein
**evm.model.Lp_chr1_0.18995 (GCTA)**	*A. tauschii*	0	92	91.49	XM_020296114.1	Protein timeless homolog
**evm.model.Lp_chr2_0.622 (GCTA)**	*T. dicocccoides*	0	82	79.78	XM_037619652.1	Putative FBD-associated F-box protein At3g50710
**evm.model.Lp_chr2_0.623 (GCTA)**	*T. dicocccoides*	0	85	8538.66	XM_037619698.1	Beta-1,4-xylosyltransferase IRX9
**evm.model.Lp_chr2_0.3894 (GCTA, MAGMA)**	*B.distachyon*	1e-141	63	82.52	XM_003562573.3	serine/arginine-rich SC35-like
**evm.model.Lp_chr2_0.3895 (GCTA)**	*B.distachyon*	1e-48	89	79.57	XM_003572657.4	S-norcoclaurine synthase.
**evm.model.Lp_chr2_0.19756 (MAGMA)**	*A. tauschii*	0	99	87.23	XM_020329468.1	uncharacterized
**evm.model.Lp_chr2_0.23247 (GCTA)**	*A. tauschii*	0	100	84.17	XM_020330933.1	codifies for AP2-like ethylene-responsive

						transcription factor AIL5
**evm.model.Lp_chr2_0.23248 (GCTA)**	-	-	-	-	-	-
**evm.model.Lp_chr3_0.8432 (MAGMA)**	-	-	-	-	-	-
**evm.model.Lp_chr3_0.8507 (MAGMA)**	*A.tauschii*	0	51	86.23	XM_020315688.1	xaa-Pro dipeptidase-like
**evm.model.Lp_chr3_0.25825 (GCTA)**	*A.tauschii*	0	96	88.79	XM_020339997.1	NAD kinase 1
**evm.model.Lp_chr3_0.25826 (GCTA, MAGMA)**	*A. tauschii*	0	97	87.16	XM_020339996.1	inactive leucine-rich repeat receptor-like protein kinase At5g48380.
**evm.model.Lp_chr3_0.3323 (GCTA)**	-	-	-	-	-	-
**evm.model.Lp_chr3_0.8509 (MAGMA)**	*Setaria virdis*	3e-46	77	74.08	XM_034736952.1	glutathione S-transferase GSTF1
**evm.model.Lp_chr3_0.9565 (MAGMA)**	*A. tauschii*	0	91	88.60	XM_020335608.1	serine incorporator
**evm.model.Lp_chr3_0.23458 (MAGMA)**	*B.distachyon*	0	98	86.21	XM_003570476.4	Uncharacterized protein
**evm.model.Lp_chr4_0.29466 (GCTA)**	-	-	-	-	-	-
**evm.model.Lp_chr4_0.29467 (GCTA)**	-	-	-	-	-	-
**evm.model.Lp_chr5_0.11861 (GCTA)**	*A. tauschii*	0	97	80.93	XM_010232430.2	F-box/LRR-repeat protein 14.
**evm.model.Lp_chr5_0.11862 (GCTA)**	*A. tauschii*	0	87	88.51	XM_020343219.1	
**evm.model.Lp_chr5_0.11920 (MAGMA)**	*B.distachyon*	0	100	93.18	XM_003577987.4	heavy metal-associated (HMA) isoprenylated plant protein (HIPP) 23.
**evm.model.Lp_chr5_0.11921 (GCTA)**	*A. tauschii*	0	100	86.72	XM_020300496.1	GTP-binding protein
**evm.model.Lp_chr5_0.16787 (GCTA)**	*Festuca arundinacea*	0	90	90.79	MH796656.1	Dehydration responsive element binding protein 1B-like protein.
**evm.model.Lp_chr6_0.596 (GCTA)**	-	-	-	-	-	-

**evm.model.Lp_chr6_0.15799 (MAGMA)**	*B. distachyon*	0	98	85.29	XM_003570583.4	Type I inositol polyphosphate 5- phosphatase 5.
**evm.model.Lp_chr6_0.15800 (GCTA, MAGMA)**	*A. tauschii*	0	100	87.02	XM_020300272.1	random slug protein 5-like isoform X1
**evm.model.Lp_chr7_0.1.1922 (GCTA)**	*A. tauschii*	0	90	78.60	XM_020304986.1	chloride intracellular channel protein 6-like
**evm.model.Lp_chr7_0.1.6143 (GCTA)**	*Sorgum bicolor*	2e-55	75	80.91	XM_002438295.2	glycine-rich cell wall structural proteins
**evm.model.Lp_chr7_0.1.11390 (GCTA)**	*B. distachyon*	1e-97	90	74.35	XM_003574454.4	codifying for a TIFY protein
**evm.model.Lp_chr7_0.1.22707 (GCTA, MAGMA)**	*A. tauschii*	0	100	91.76	XM_020344125.1	cytochrome P450 77A3
**evm.model.Lp_chr7_0.1.19634 (GCTA)**	*A. tauschii*	0	96	90.76	XM_020291380.1	peroxidase P7-like
**evm.model.Lp_chr7_0.1.19642 (GCTA)**	*A. tauschii*	0	100	91.76	XM_020344125.1	

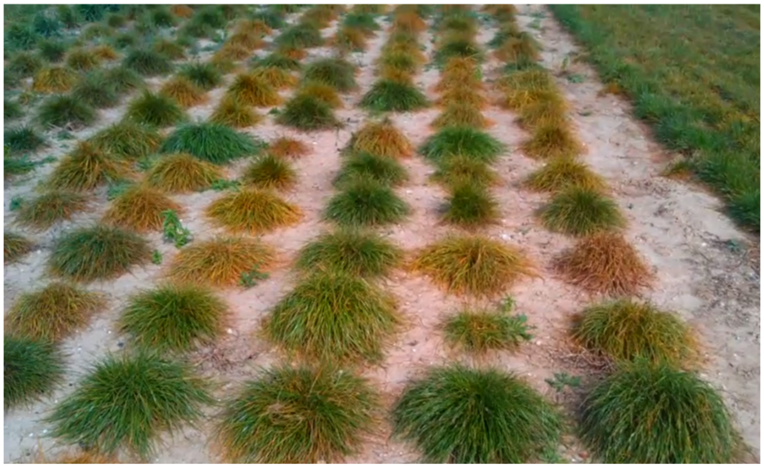

Figure S5. Symptoms of crown rust infection of perennial ryegrass.
